# Healthcare use in patients with cardiovascular disease and depressive symptoms – The impact of a nurse-led internet-delivered cognitive behavioural therapy program. A secondary analysis of a RCT

**DOI:** 10.1016/j.invent.2023.100696

**Published:** 2023-12-05

**Authors:** Ghassan Mourad, Johan Lundgren, Gerhard Andersson, Peter Johansson

**Affiliations:** a Department of Health, Medicine and Caring Sciences, Linköping University, Linköping, Sweden; b Department of Behavioural Sciences and Learning, Linköping University, Linköping, Sweden; c Department of Biomedical and Clinical Sciences, Linköping University, Linköping, Sweden; d Department of Clinical Neuroscience, Karolinska institute, Stockholm, Sweden; e Department of Internal Medicine in Norrköping, Department of Health, Medicine and Caring Sciences, Linköping University, Linköping, Sweden

**Keywords:** Cardiovascular disease, Depressive symptoms, Healthcare use, Hospital admissions, Outpatient services, Internet-delivered cognitive behavioural therapy

## Abstract

**Background:**

Depressive symptoms in patients with cardiovascular disease (CVD) can lead to increased healthcare use. In a randomized controlled trial (ClinicalTrials.gov, NCT02778074), we reported that a 9-week internet-delivered cognitive behavioural therapy (iCBT) program (*n* = 72) compared to an online discussion forum (ODF) (n = 72) had moderate to large effect on depression in CVD outpatients. In this secondary analysis, we aimed to describe and compare the effect of iCBT compared to ODF regarding healthcare use and to identify factors impacting healthcare use in these groups.

**Methods:**

Data on healthcare use were retrieved from care data registries in five hospitals in Southeastern Sweden.

**Results:**

The year prior to intervention, the iCBT group had a mean of 31 outpatient clinic/primary care contacts per patient compared with 21 contacts the year after. The corresponding numbers for the ODF group were 37 and 25. The decrease was 32 % in both groups and did not differ significantly (*p* = 0.261 and *p* = 0.354) between the groups. Regarding hospital admissions, the iCBT group had 0.8 admissions per patient the year before and 0.6 the year after the intervention, a decrease by 25 %, whereas the ODF group had 1.1 and 0.6 admissions respectively, a decrease by 45 %. The difference was not statistically significant *(p* *=* *0.270 and p* *=* *0.883)* between the groups. Improvement in depressive symptoms post intervention were significantly (Beta = 0.459, *p* = 0.047) associated with a decrease in number of outpatient contacts in the iCBT group. In the ODF group, better mental health-related quality of life post intervention was significantly (Beta = −0.429, *p* = 0.045) associated with a decrease in number of hospital admissions.

**Conclusion:**

Reduced depressive symptom scores following intervention were associated with lower outpatient service use, but iCBT was not superior compared to ODF. This implicates that reducing depression in CVD patients, regardless of the type of internet-delivered intervention used, is important since it may reduce healthcare use in these patients.

## Highlights

• Patients with CVD use outpatient healthcare services to a great extent.
• Both iCBT and online discussion forum gave a decrease in healthcare use by one-third.
• Reduced depressive symptom scores were associated with lower outpatient service use.
• iCBT was not superior to online discussion forum in decreasing healthcare use.

## 1 Introduction

Depression is common (20 %–40 %) in patients with cardiovascular disease (CVD) and significantly worsens the health of the patients (Hare et al., 2014). Patients with CVD and depression have a shorter life expectancy (Johansson et al., 2013) and higher risk of non-fatal cardiac events (e.g. re-hospitalization) than CVD patients without depression (Davydow et al., 2015). Thus, depression adds an extra burden for patients in addition to the consequences of CVD.

Depression also has a societal impact. Studies show that depression increases the use of healthcare services in patients with or without various chronic somatic diseases (Tusa et al., 2019; Sporinova et al., 2019). In a study focusing on CVD patients, depression was found to be a significant predictor of increased healthcare use and costs (Curcio et al., 2019; Szpakowski et al., 2017). These costs were related to healthcare use in almost all healthcare sectors, including acute inpatient care, chronic care, physician costs and medications (Szpakowski et al., 2017). Palacios et al. (2018) investigated depression and healthcare costs in outpatients with coronary heart disease over a three-year period. They found that those with worsening or chronic depressive symptoms had over twice the cost than patients with no depressive symptoms. In addition, patients with worsening or chronic depressive symptoms that were detected and treated had lower costs compared to those that were undetected and untreated (Palacios et al., 2018). Depressed CVD patients experience poorer health-related quality of life (HRQoL) (Hare et al., 2014), but although there is a strong relationship between depressive symptoms and HRQoL, HRQoL mirrors a broader perspective of the CVD patient's health than the mental aspects, such as depressive symptoms. This suggests that interventions aiming to decrease depression in patients with CVD, particularly outpatients who have worsening or chronic depressive symptoms, also could improve HRQoL, reduce the burden for the patient, as well as the costs for society. Palacios et al. (2018) emphasized that such interventions should target outpatients rather than those hospitalized or newly discharged. Furthermore, such interventions should focus more on the effects on depression and healthcare costs than the impact on adverse cardiac events or prognosis.

Internet-delivered cognitive behavioural therapy (iCBT) has proved to be effective in patients with depression both in short- and long-term follow-up (Furukawa et al., 2021; Andersson et al., 2018), and also as effective as face-to-face treatment (Hedman-Lagerlöf et al., 2023). Regarding the health-economic benefits of iCBT for depression, Donker et al. (2015) were not able to perform a meta-analysis of 16 iCBT studies as the studies used different methods in their economic evaluation. The authors however stated that iCBT appeared to be cost-effective, especially the guided intervention modalities. Kolovos et al. (2018) were able to evaluate cost-effectiveness in a meta-analysis of five iCBT studies on depression. Surprisingly, they reported similar cost-effectiveness of iCBT compared to control conditions, such as care as usual and psychoeducation or care as usual and a self-help booklet or a waiting list. The authors commented that the five studies only constituted a small fraction of the number of randomized controlled iCBT studies published (Kolovos et al., 2018). Thus, in the literature, there is a knowledge gap regarding health-economic evaluations of iCBT and particularly the impact of iCBT on healthcare use (Pedersen et al., 2023).

The number of iCBT studies on patients with chronic somatic illnesses, such as those with CVD, is still limited both regarding the effect on depression and health-economy (Reavell et al., 2018; McCombie et al., 2015). Patients with chronic somatic conditions and depression use healthcare resources to a great extent, and therefore the impact of iCBT on healthcare use is of particular interest. A previous research paper by the current authors (Johansson et al., 2019), reported that a 9-week iCBT program consisting of seven modules including goal setting, psychoeducation, problem solving, and behavioural activation was compared to an online discussion forum (ODF) where new discussion topics regarding their heart disease were presented each week over a nine-week period. iCBT showed to have moderate to large effects on depressive symptoms and HRQoL in CVD outpatients, although the associations between healthcare use and depressive symptoms were not reported in that study. The aim of this study was therefore to describe and compare the effect of iCBT compared to ODF regarding healthcare use and to identify factors impacting healthcare use in these groups.

## 2 Methods

### 2.1 Ethical approval and consent to participate

The study was carried out in accordance with the Declaration of Helsinki and was approved by the Regional Ethical Review Board in Linköping, Sweden (ref. no. 2016/72-31). The primary study was registered at ClinicalTrials.gov (NCT02778074). Healthcare use was not pre-specified in the study protocol as one of the secondary analyses but was so in the ethical approval. Participants signed an informed consent before study inclusion.

### 2.2 Study design and research participants

The present study is a secondary analysis of a randomized controlled trial (RCT) reporting a detailed description of healthcare use and differences between CVD patients enrolled to either iCBT or ODF. The primary study with the primary aim to reduce depressive symptoms has been described in detail elsewhere (Johansson et al., 2019). Briefly, invitations were sent by post to all patients who had been in contact with the medical or cardiac clinics at five hospitals in Southeastern Sweden. Information on these patients was provided from data registries at these hospitals. After screening for depressive symptoms (i.e., Patient Health Questionnaire-9 (PHQ-9) score ≥ 5), 144 patients who fulfilled the criteria (e.g. age above 18 years, and not being hospitalized the past four weeks prior to inclusion) and consented to participate were allocated to nine weeks of iCBT (*n* = 72) or ODF (n = 72). Of the 72 patients in the ODF group, 27 (38 %) received iCBT after study completion. The iCBT program was tailored to patients with CVD and guided by nurses with clinical experience of CVD and psychiatry and a brief education in iCBT. The iCBT program comprised one goal setting module, two psycho-education modules, one problem-solving module, two behavioural activation modules and a summary module. All modules included homework assignments and written feedback was provided on each assignment from a study nurse. A total of 60 % of those in the iCBT group completed all seven modules, and 82 % completed at least 4 modules. The ODF consisted of nine discussion topics moderated by a nurse. In this group, 27 % were active at least nine times during the nine weeks of the intervention.

### 2.3 Data collection

Data regarding depressive symptoms and HRQoL were obtained at baseline and at 9-weeks follow-up in the main RCT study (Johansson et al., 2019). Data on healthcare use comprised all healthcare contacts within primary care, outpatient clinics and hospital care, one year prior to study start and one year after end of the study, which lasted for 9 weeks. Retrieved data was categorized into outpatient and inpatient data. Data on healthcare use were not possible to obtain for six participants (4 %). This was because they were residents in other regions and data could therefore not be accessed, despite receiving treatment for CVD in any of the four recruitment hospitals and thereby included in the RCT.

### 2.4 Research instruments

Depressive symptoms were measured using PHQ-9, which consists of nine items that are rated on a four-point scale from (not at all, several days, more than half the days, nearly every day) and with a summary score ranging from 0 to 27 (Spitzer et al., 1999). Scores between 0 and 4 indicate no depressive symptoms, 5–9 mild depressive symptoms; 10–14 moderate depressive symptoms and scores of 15 and above indicate severe depressive symptoms. The PHQ-9 has demonstrated good psychometric properties (Kroenke et al., 2001; Spitzer et al., 1999), also in patients with CVD (i.e. heart failure) (Hammash et al., 2013). The PHQ-9 has also proved to be valid in the computerized format (Erbe et al., 2016).

The Short Form 12 (SF-12) (Ware Jr. et al., 1996) and the EuroQol Visual Analogue Scale EQ-VAS (Brooks et al., 1991) were used to assess HRQoL. The SF-12 comprises 12 items that originates from the longer instrument Short Form-36 (SF-36) (Ware Jr. et al., 1996), and has been used in a wide range of studies and populations, including patients with CVD (Müller-Nordhorn et al., 2004). The SF-12 is divided into a physical component score (PCS) and a mental component score (MCS). The EQ-VAS is a visual analogue scale that ranges between 0 (worst health you can imagine) and 100 (best health you can imagine).

Data on healthcare use were collected from care data registries in Region Östergötland, Region Jönköping County and Region Kalmar County, Sweden.

### 2.5 Data analysis

Data on healthcare use were summed for each participant and divided into contacts and admissions one year prior to study start, and one year after end of study (i.e., the 9 week intervention period was not included in the analysis). Length of hospital stay was reported in days, but due to different registration procedures at the different hospitals, where some reported the exact time for the hospital stay and some only reported the dates, each admission was reported as at least one day even when it was not 24 h.

Demographic data and healthcare use were presented using numbers, percentages, mean values, and standard deviations. Differences in demographic data between the iCBT and ODF groups were analyzed using Chi-Square test for categorical variables and Student's *t*-test for continuous variables. Student's *t*-test was also used to compare the iCBT and ODF groups with regard to healthcare use (i.e., number of outpatient clinic and/or primary care contacts, and/or hospital admissions, and/or length of stay). For comparison within groups between the year prior to and the year post intervention, paired samples *t*-test was used as data were continuous.

Pearson correlations as well as multiple linear regression were used to explore possible associations between change in healthcare use and depressive symptoms and HRQoL. All variables (See Table 1, Table 2) that correlated significantly with change in healthcare use were used as covariates in the linear regression analysis (i.e., sex, marital status, occupational status, financial situation, and number of co-morbidities). However, since change scores for depressive symptoms and HRQoL do not automatically indicate a significant improvement in depressive symptoms and HRQoL (for example, a patient can have decreased PHQ-9 scores from 25 to 20, but still having a high level of depressive symptoms), we used the follow-up scores for depressive symptoms and HRQoL as variables in the regression analysis.

**Table 1:** Demographic data of patients randomized to Internet-delivered cognitive behavioural therapy (iCBT) or online discussion forum (ODF).

	iCBT (*n* = 72)	ODF (n = 72)
Sex, n (%)^a^		
Male	47 (65)	42 (58)
Female	25 (35)	30 (42)
Age, mean (SD)^a^	61 (13)	64 (12)
Educational level, n (%)^a^		
Elementary	7 (10)	12 (17)
Upper secondary/high school	16 (22)	21 (29)
Post-secondary vocational education	12 (17)	6 (8)
College/university	37 (51)	33 (46)
Work status, n (%)^a^		
Working	26 (36)	18 (25)
Sick leave/disability pension	8 (11)	10 (14)
Retired	32 (44)	36 (50)
Other	6(8)	8 (11)
Marital status, n (%)^a^		
Married/cohabiting	53 (74)	53 (74)
Living alone	19 (26)	19 (26)
Financial Situation, n (%)^a^		
Very good	10 (14)	11 (15)
Good	49 (68	45 (63)
Problematic	10 (14)	14 (19)
Very problematic	3 (4)	2 (3)
Smoking, n (%)^a^		
Never	33 (46)	36 (50)
Ex-smoker	37 (51)	33 (46)
Smoker	2 (3)	3 (4)
Alcohol, n (%)^a^, ^b^		
0–4 units per week	51 (71)	58 (80)
5–9 units per week	17 (24)	10 (14)
10–14 units per week	3 (4)	4 (6)
15 or more units per week	1 (1)	0 (0)
Comorbidities, n (%)^a^		
Ischemic heart disease	34 (47)	29 (40)
Atrial fibrillation	40 (56)	41 (57)
Heart failure	18 (25)	20 (28)
Hypertension	36 (50)	40 (56)
Diabetes	8 (11)	13 (18)
Pulmonary disease	7 (10)	8 (11)
Stroke and/or TIA	9 (13)	10(14)
Renal disease	3 (4)	2 (3)
Cancer	7 (10)	9 (13)
Other psychiatric disorder	4 (6)	8 (11)
Co-morbidities (3 or more), n (%)	10 (14)	13 (18)

TIA = Transitory Ischemic Attack.

a No statistically significant differences were found between the groups.

b Equivalent of 12 g of alcohol.

**Table 2:** Depressive symptom and HRQoL scores at baseline and 9-week follow-up in the patients randomized to Internet-delivered cognitive behavioural therapy (iCBT) or online discussion forum (ODF).

	iCBT (n = 72)		ODF (n = 72)		*p*-value⁎	Effect size (Cohen *d*)
	Baseline mean (SD)	9-Weeks mean (SD)	Baseline mean (SD)	9-Weeks mean (SD)		
PHQ-9	10.7 (4.5)	6.6 (4.8	10.2 (5.1)	8.7 (4.6)	<0.001	0.62
EQ-VAS	53.3 (20.0)	65.1 (21.8)	57.2 (18.1)	57.0 (22.1)	<0.001	0.62
PCS12	39.7 (10.1)	41.7 (10.6)	37.6 (11.0)	37.8 (11.6)	0.06	0.32
MCS12	35.9 (9.2)	43.4 (11.0)	36.4 (10.0)	38.0 (10.5)	<0.001	0.66

⁎ The *p*-values represent the difference in the change score between groups.

The independent variables used were all entered in the regression model. The associations were classified as weak with beta values of 0.10–0.30, moderate with beta values of 0.31–0.50, and as strong with beta values above 0.50. All analyses in the present study are based on the collected data, without imputation due to little missing data. The level of <0.05 was set for significance. The IBM SPSS version 25.0 was used for data analysis.

## 3 Results

### 3.1 Study participants

A detailed presentation of the study participants is presented in the main publication (Johansson et al., 2019). Shortly described, we enrolled participants from medical and cardiology clinics at five hospitals in the southeastern region of Sweden. Invitation letters were sent via mail to individuals with at least one diagnosis of atrial fibrillation or atrial flutter (International Classification of Diseases (ICD) codes I48. or I49.), coronary heart disease (ICD codes I20. or I25.), and heart failure (ICD codes I42. or I50.), who had undergone at least one outpatient visit or hospitalization in the year preceding recruitment. A total of 11,992 patients being contacted. Those interested in participating in the study were directed to register on the study website (www.dohart.se). Out of 272 patients who registered their interest in study participation, 144 (53 %) fulfilled the inclusion criteria and were randomized to either iCBT (*n* = 72) or ODF (n = 72), see Table 1. The iCBT group had a mean age of 61 ± 13 years compared to 64 ± 12 years in the ODF group. Both iCBT and ODF groups consisted of more men (65 % and 58 %) than women (35 % and 42 %). About half of both groups had a college/university degree, and three out of four were married or cohabiting. There were no statistically significant differences between the two groups regarding any of the demographic variables.

Depressive symptoms and HRQoL scores at baseline and 9-weeks follow-up have been presented in the main publication (Johansson et al., 2019), see Table 2.

### 3.2 Healthcare use in patients randomized to iCBT or ODF

#### 3.2.1 Outpatient clinic and/or primary care contacts

Table 3 shows the number of outpatient clinic and/or primary care contacts in the iCBT and ODF groups one year prior to and one year post intervention. In the iCBT group, there was a decrease per patient from 31 contacts the year prior to intervention to 21 contacts the year after. The corresponding numbers of contacts were 37 and 25 contacts for the ODF group.

**Table 3:** Outpatient clinic and/or primary care contacts in patients randomized to internet-delivered cognitive behavioural therapy or online discussion forum.

	iCBT (*n* = 70)	ODF (*n* = 68)	Between group differences prior to and post intervention, *p*-value
Number of contacts per patient, mean (SD)			
1 year prior to intervention	31 (31)	37 (31)	0.261
1 year post intervention	21 (25)	25 (23)	0.354
Within group differences prior to and post intervention, p-value	<0.001	<0.001	

**Fig. 1:**
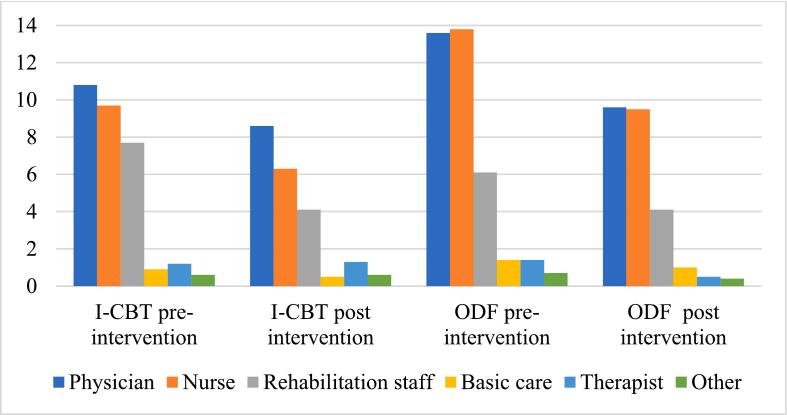
Type of outpatient contacts in patients randomized to iCBT or ODF one year prior to and one year post intervention.

This gave a decrease by 32 % in both groups. Thus, no statistically significant between-group differences were found in the number of outpatient clinic and/or primary care contacts the year post intervention compared to the year prior to intervention. Most outpatient clinic/primary care contacts were made to physicians, nurses, and rehabilitation staff (i.e., physiotherapists and occupational therapists), see Fig. 1. There were no statistically significant differences between the groups in any of the types of contacts. Within-group analysis revealed a statistically significant (*p* < 0.001) decrease between the years in both groups.

#### 3.2.2 Hospital admissions and length of stay

Table 4 presents the number of hospital admissions in the iCBT and ODF groups one year prior to and one year post intervention. In the iCBT group, there was a decrease per patient from 0.8 hospital admissions the year prior to intervention to 0.6 admissions the year after. The ODF group had a decrease from 1.1 to 0.6 admissions between the years. The iCBT group had a decrease by 25 %, compared to 45 % in the ODF group. However, the difference between the groups was not statistically significant. The decrease in the number of admissions was statistically significant in the ODF group (*p* = 0.03), but not in the iCBT group.

**Table 4:** Hospital admissions in patients randomized to internet-delivered cognitive behavioural therapy or online discussion forum.

	iCBT (n = 70)	ODF (n = 68)	Between group differences prior to and post intervention, *P*-value
Number of admissions per patient, mean (SD)			
1 year prior to intervention	0.8 (1.5)	1.1 (1.6)	0.270
1 year post intervention	0.6 (1.5)	0.6 (2.2)	0.883
Within group differences prior to and post intervention, *p*-value	0.307	0.030	

In the iCBT group, the length of stay decreased by 13 %, i.e., a change from 2.4 days to 2.1 days per patient (Table 5), whereas in the ODF group, there was a decrease by 56 %, i.e., a change from 4.1 days to 1.7 days. The difference between the groups was not statistically significant.

**Table 5:** Length of stay in days in patients randomized to internet-delivered cognitive behavioural therapy or online discussion forum.

	iCBT (n = 70)	ODF (n = 68)	Between group differences prior to and post intervention, *P*-value
Length of stay in days per patient, mean (SD)			
1 year prior to intervention	2.4 (5.9)	4.1 (8.9)	0.173
1 year post intervention	2.1 (9.3)	1.7 (6.4)	0.776
Within group differences prior to and post intervention, *p*-value	0.830	0.052	

### 3.3 The association between depressive symptoms and changes in healthcare use in patients randomized to iCBT or ODF

The factors associated with change in healthcare use (i.e., outpatient clinic and/or primary care contacts, and/or hospital admissions) the year post intervention compared to the year before are presented for the iCBT and ODF group respectively in Table 6. After adjustments for the other correlates of change in healthcare use, lower PHQ-9 scores in the iCBT group, post intervention demonstrated a statistically significant moderate association (Beta = 0.459, *p* = 0.047), with a decrease in the number of outpatient clinic and/or primary care contacts. In the ODF group, higher MCS12 scores post intervention had a statistically significant moderate association (Beta = −0.429, *p* = 0.045) with a decrease in number of hospital admissions.

**Table 6:** Factors impacting healthcare use (outpatient clinic/primary care contacts, and hospital admissions) in patients receiving internet-delivered cognitive behavioural therapy (iCBT) or online discussion forum (ODF).

Explanatory variables	iCBT (*n* = 70)				ODF (*n* = 68)			
	Change in contacts between years		Change in admissions between years		Change in contacts between years		Change in admissions between years	
	Beta	p-value	Beta	p-value	Beta	p-value	Beta	p-value
Sex	−0.029	0.835	0.104	0.476	0.182	0.193	0.002	0.989
Marital status	0.026	0.862	−0.063	0.689	−0.283	0.067	−0.253	0.093
Occupation	−0.083	0.602	0.124	0.456	0.009	0.952	0.205	0.165
Financial situation	−0.271	0.081	−0.053	0.743	0.003	0.983	0.166	0.224
Number of co-morbidities	0.075	0.597	−0.102	0.490	0.152	0.330	0.051	0.739
PHQ-9 score post intervention	0.459	**0.047**	0.158	0.507	−0.229	0.304	−0.282	0.197
PCS12 score post intervention	0.015	0.937	0.115	0.576	−0.081	0.745	−0.298	0.226
MCS12 score post intervention	0.181	0.388	−0.085	0.699	−0.288	0.183	−0.429	**0.045**
EQ-VAS score post intervention	0.272	0.185	−0.018	0.934	0.341	0.169	0.439	0.072

## 4 Discussion

To our knowledge, studies performing health-economic evaluations of iCBT interventions on depression in patients with chronic somatic states are lacking. In this study, we aimed to describe and compare the iCBT and ODF groups with regard to healthcare use and to identify factors impacting healthcare use in these groups. We found that both the iCBT and ODF groups had a high level of healthcare use (especially outpatient clinic/primary care contacts) the year prior to the intervention. During the year post intervention, the healthcare use had decreased by approximately one third in both groups. However, this was not significantly different between the groups. Interestingly in the iCBT group only, lower levels of depressive symptoms post intervention were associated with a decrease in the number of outpatient clinic and/or primary care contacts.

In the current study, we found that the use of outpatient services the year prior the intervention (31 for iCBT and 37 for ODF) was higher in both groups than was reported for CVD patients in a previous cross-sectional study by Mourad et al. (2012). In that study, patients with myocardial infarction had 10 contacts and those with Angina Pectoris had 19 healthcare contacts, and depressive symptoms were found in about 25 % of the cases. In the current study, we did not measure depression the year prior to intervention, but as indicated in Palacios et al. (2018), depression in CVD patients can become chronical since depression seldom is detected and patients are therefore not offered treatment (Palacios et al., 2018). A possible explanation behind the high use of outpatient services the year prior the intervention in our sample could therefore be due to depression. The association between mental health disorders and increased healthcare use among patients with chronic diseases has been noted in previous research (Sporinova et al., 2019). It is also known that the addition of depression to CVD carries a higher risk for healthcare use, in particular for those with chronic depression (Palacios et al., 2018). Morrissey (2019) found in a population-based study of 791 individuals with CVD that the addition of a non-related comorbidity (such as depression/anxiety) led to a three times higher risk for use of outpatient services, such as general practitioner visits, and 1.5 times higher risk for use of inpatient services. In our study, most of the contacts within outpatient/primary care clinics were to physicians and primary care nurses. Hence, as depression is one of the most important factors associated with increased healthcare use in CVD patients, this indicates a need to optimise the management of depression in these patients. When performing interventions that target depression in CVD patients, Palacios et al. (2018) suggested that these should focus the impact on depression and healthcare use in CVD outpatients.

We have previously reported that 9 weeks of iCBT compared to ODF (i.e., an active control group) had significantly better effects on depressive symptoms and HRQoL in CVD outpatients (Johansson et al., 2019). In the present study we reported the effect on healthcare use. The number of admissions was relatively low in both groups, before as well as after the intervention, and it is therefore hard to identify any differences between the groups. The number of hospital admissions was 0.8 in the iCBT and 1.1 in the ODF groups prior the intervention, and the year post intervention this had decreased to 0.6 in both groups. The low number of hospital admission, especially in the iCBT group may also explain why only a statistically significant decrease was detected in the ODF group. We found that both groups had a statistically significant decrease in number of outpatient clinic and/or primary care contacts the year post intervention by approximately 30 %, but no differences could be found between the groups (Table 3). The difference between numbers of in- and outpatient service use is mostly due to the structure of the Swedish healthcare system, were primary care since long time ago serves as the first check point and people are initially referred there for consultation regarding their care needs (Joumard and Organisation for Economic Co-operation and, 2010). Since patients are referred to primary care, this suggests that interventions targeting depressive symptoms in CVD are more likely to have impact on the number of outpatient clinic and/or primary care contacts than the number of hospital admission.

We could not find that iCBT was superior to ODF in decreasing the use of healthcare services. A similar finding was reported in the meta-analysis by Kolovos et al. showing that iCBT compared to control was not more cost-effective (Kolovos et al., 2018). Still, the finding that iCBT at first sight does not seem to have any health-economic benefits may be related to the design of the control conditions. In our study iCBT was compared to an active control group (i.e., ODF). After completion of ODF, all patients were offered iCBT. This design was due to ethical reasons, and 38 % of them accepted and received iCBT. In the meta-analysis some of the five studies also used more active control approaches such as psychoeducation, care as usual and a self-help booklet or a waiting list and who also received iCBT (Kolovos et al., 2018).

In our study, patients in the control group were involved in a discussion forum that enabled them access to other CVD patients´ experiences, tips, and support, and hypothetically also a reduced need of support from healthcare services. Other studies (Enck and Zipfel, 2019) also show that active control approaches have impact on depressive symptoms and makes it harder for iCBT to demonstrate a greater impact on healthcare use. As mentioned, those in the ODF group also received iCBT. This could explain why both iCBT and ODF groups showed a decrease by 30 % in healthcare use. Having in mind that most CVD patients with depression in real life are not detected or receive any treatment, Celano et al. (2018) argues that iCBT in CVD patients would lead to health-economic benefits.

We also demonstrated that lower depressive symptoms scores post intervention were associated with a decrease in the number of outpatient clinic and/or primary care contacts, in favour of the iCBT group. This indicates that lower levels of depressive symptoms, that in a significantly higher extent were achieved by the iCBT group compared to ODF (Johansson et al., 2019), resulted in a decrease in outpatient service use. In the ODF group, improved HRQoL as measured by MCS12 scores post intervention was moderately associated with a decrease in number of hospital admissions. This association was not found in the iCBT group. Since impaired HRQoL has been associated with increase in hospital admissions in CVD (Pocock et al., 2021), the improved HRQoL in the ODF group might explain the statistically significant decrease of 45 % in hospital admissions.

Since our control group was an active control and of which one-third also received iCBT post-intervention, we cannot exclude the possibility that iCBT has health-economic benefits and may have reduced the differences between the groups regarding healthcare use. Based on the intervention group including 72 patients, the time consumption for a therapist in the iCBT program was about 13 min per patient per week, which sums up to 2 h per patient for the nine weeks treatment and to 140 h for the whole iCBT group. Face-to-face CBT is normally at least 10 sessions/weeks with about 45 min per session. The time needed for treating 72 patients would therefore sum up to 540 h for the whole treatment. By using the Internet to deliver the CBT treatment, the time saving would correspond to about 400 therapist hours. Above this, there are other savings in relation to the patients and society in general since the treatment can be performed when suitable for the patients without travel expenses to healthcare facilities or lost working hours for therapist appointments. Thus, iCBT for depressive symptoms in CVD seems to be cost-saving, but in order to draw the correct conclusions, a cost-effectiveness analysis needs to be carried out.

### 4.1 Limitations

The study was primarily designed to improve depressive symptoms and hence not to measure differences in healthcare use or to compare healthcare use depending on type of CVD. Therefore, these results should be interpreted with caution. To detect a statistically significant difference between the groups with a power of 80 % based on our values regarding outpatient clinic/primary care contacts, would have required a sample size of 567 patients per group. After completion of ODF, 38 % of the patients received iCBT, and this might have impacted healthcare use positively and made it difficult to detect differences between groups. It would have been informative to know more about the reason (e.g., CVD, mental health) for the healthcare use, but our data registers did not include such information for all healthcare use. To gain such information, scrutinizing patients´ medical records would have been needed, which would have required permissions from the authorities that can be difficult to obtain, as well as a great amount of working time. Our CVD population consisted of more males than females. This reflects a normal CVD population in the age group that we have managed to recruit as more men than women develop CVD at a younger age. On the other hand, a large proportion of our patients had a college/university degree, which may not be representative for an average CVD population. It is possible that iCBT is more likely to attract and suit people with higher education. This may impact the generalizability of such interventions among CVD patients with lower education. Despite these limitations, we believe that the results provide important information since there is a lack of studies performing health-economic analyses of iCBT in CVD patients with depressive symptoms.

## 5 Conclusions

We found high levels of outpatient healthcare use in the iCBT and ODF groups the year prior to the intervention. One year after the intervention, outpatient healthcare use had decreased by one third in both groups. Even though lower levels of depressive symptoms post intervention in the iCBT group were associated with a decrease in outpatient healthcare use, there was no difference between the iCBT and ODF groups regarding healthcare use. This is most likely because the study was underpowered. From a health economic perspective, this suggests that for clinical practice, reducing depression in CVD patients could be important. This is regardless of the type of internet-delivered intervention used, as it may lead to decreased healthcare use in these patients.

## Abbreviations

CVD: cardiovascular disease
EQ-VAS: EuroQol Visual Analogue Scale
HRQoL: health-related quality of life
iCBT: Internet-delivered cognitive behavioural therapy
MCS: mental component score
ODF: online discussion forum
PHQ-9: Patient Health Questionnaire-9
PCS: physical component score
RCT: randomized controlled trial
SF-12: Short Form 12;
SF-36: Short Form-36

## Declaration of competing interest

The authors report there are no competing interests to declare.

## Data Availability

The data that support the findings of this study may be made available from the corresponding author upon reasonable request.
